# Cost-effectiveness of preoperative biliary drainage for obstructive jaundice in pancreatic and periampullary cancer

**DOI:** 10.1016/j.jss.2014.07.060

**Published:** 2015-01

**Authors:** Stephen Morris, Kurinchi S. Gurusamy, Jessica Sheringham, Brian R. Davidson

**Affiliations:** a Department of Applied Health Research, University College London, London, UK; b Research Department of General Surgery, Royal Free Hospital, University College London Medical School, London, UK

**Keywords:** Preoperative biliary drainage, Periampullary cancer, Obstructive jaundice, Cost-effectiveness analysis

## Abstract

**Background:**

A recent Cochrane Review found that preoperative biliary drainage (PBD) in patients with resectable pancreatic and periampullary cancer undergoing surgery for obstructive jaundice is associated with similar mortality but increased serious morbidity compared with no PBD. Despite this clinical evidence of its lack of effectiveness, PBD is still in use. We considered the economic implications of PBD *versus* direct surgery for obstructive jaundice in patients with pancreatic and periampullary cancer.

**Materials and methods:**

Model-based cost-utility analysis estimating mean costs and quality-adjusted life years (QALYs) per patient from the perspective of the UK National Health Service over a 6-month time horizon. A decision tree model was constructed and populated with probabilities, outcomes, and cost data from published sources. One-way and probabilistic sensitivity analyses were undertaken.

**Results:**

PBD was more costly than direct surgery (mean cost per patient £10,775 [$15,616] *versus* £8221 [$11,914]) and produced fewer QALYs (mean QALYs per patient 0.337 *versus* 0.343). Not performing PBD would result in cost savings of approximately £2500 ($3623) per patient to the National Health Service. PBD had <10% probability of being cost-effective at a maximum willingness to pay for a QALY of £20,000 ($28,986) to £30,000 ($43,478).

**Conclusions:**

There are significant cost savings to be gained by avoiding routine PBD in patients with resectable pancreatic and periampullary cancer where PBD is still routinely used in this context; this economic evidence should be used to support the clinical argument for a change in practice.

## 1 Introduction

Obstructive jaundice is a common symptom in patients with periampullary cancer (located near the ampulla of Vater) or cancer of the pancreatic head. Surgical resection is the only option for cure [1–3]. Because obstructive jaundice is thought to increase the risk of developing postoperative complications, preoperative biliary drainage (PBD) was introduced to improve the postoperative outcome [4]. It has since been incorporated into the standard surgical treatment algorithm of periampullary cancer and cancer of the pancreatic head in the majority of hospitals [4]. Other factors that may influence the use of PBD include temporary contraindications for surgery such as severe malnutrition and other comorbidities that have to be treated before surgery and the interval between diagnosis and treatment. If there is a long waiting time before surgery, PBD may have to be performed [1]. However, the wisdom of delaying surgery in people with an aggressive cancer such as pancreatic cancer is questionable.

In several studies, PBD reduced morbidity and mortality after surgery [4–7]. However, a recent Cochrane Review of the six randomized clinical trials evaluating the safety and effectiveness of PBD *versus* no PBD found that PBD in patients undergoing surgery for obstructive jaundice is associated with similar mortality but increased serious morbidity compared with no PBD [8,9]. The review concluded that PBD should not be used routinely. Nonetheless, there is evidence that PBD is still commonly used in this context [10] suggesting that clinical considerations alone are not sufficient to change practice. Consideration of the economic implications of carrying out routine PBD to health systems may be needed.

A review of the National Health Service (NHS) Economic Evaluations Database [11] using the search term “biliary drainage” identified eight studies, but none of these evaluated PBD *versus* direct surgery for obstructive jaundice in patients with pancreatic and periampullary cancer. Therefore, this study investigates the cost-effectiveness of PBD *versus* direct surgery for obstructive jaundice in patients with pancreatic and periampullary cancer.

## 2 Materials and methods

This is a model-based cost-utility analysis to estimate the mean cost per patient and the mean outcome per patient associated with PBD *versus* direct surgery for obstructive jaundice in patients with pancreatic and periampullary cancer. The outcome measure is quality-adjusted life years (QALYs), which combine length of life and quality of life [12]. QALYs are the recommended outcome for use in economic evaluations in the United Kingdom as they are a common unit that allow for comparable decisions about resource allocation across different health conditions.

The analysis is undertaken from the perspective of the UK NHS. Costs are calculated in 2011–2012 UK£ with US$ given in parentheses (UK1 = US$1.449). Since treatment for an acute condition is being investigated and the Cochrane Review found that PBD had no impact on mortality, a time horizon of 6 mo for costs and outcomes was considered to be appropriate and discounting of costs and benefits was unnecessary.

### 2.1 Model structure

**Fig. 1:**
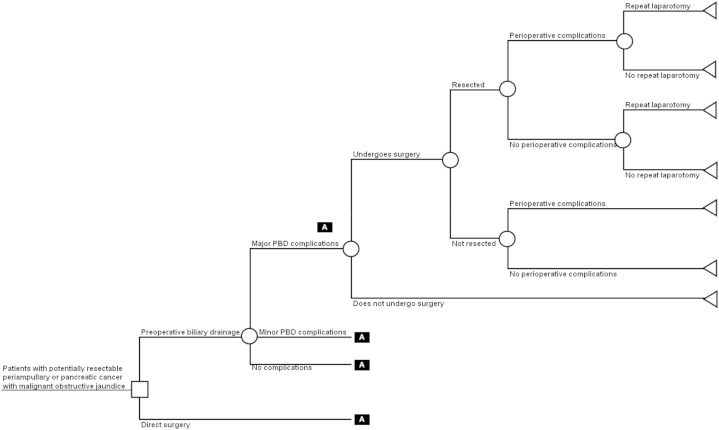
Decision tree model structure.

The analysis uses a decision tree to describe the options being compared and the possible pathways following them (Fig. 1). This is a commonly used approach in cost-effectiveness studies of health care programs [12]. The nodes of a decision tree are points where more than one event is possible. The branches are mutually exclusive events following each node. Decision nodes, represented by squares, show the different options that might be chosen by decision makers based on the costs and benefits they produce (e.g., to perform PBD or not). Chance nodes, represented by circles, show uncertain events, each of which is associated with a probability that it will occur (e.g., whether or not PBD will have major, mild, or no complications). Terminal nodes, represented by triangles, are the endpoints of a decision tree, beyond which no further pathways are available. Each terminal node has costs and QALYs associated with it, summarizing the sequence of decisions and events on a unique path leading from the initial decision node to that terminal node. These costs and QALYs are expected values based on the probability of each event on the pathway occurring up to that point, and the costs and QALYs associated with each event.

Patients enter the model with potentially resectable periampullary or pancreatic cancer with malignant obstructive jaundice. If they undergo PBD, the procedure may have major, minor, or no complications. In any case, patients may or may not undergo surgery subsequently because of the complications of PBD or the underlying cancer, and a proportion of patients undergoing surgery will be resected. Those who undergo surgery may experience perioperative complications, and a proportion of those who are resected may require a repeat laparotomy for recurrence or long-term complications such as adhesions.

For patients undergoing surgery directly, without PBD, it was assumed that the treatment pathway is the same as the one subsequent to PBD, but the probabilities, costs, and QALYs associated with each pathway may be different.

### 2.2 Probabilities

The probabilities associated with mutually exclusive events at each chance node were obtained from published sources (Tables 1 and 2) [13–18]. Additional data were extracted from the six randomized clinical trials included in the Cochrane Review on the probability of major and minor complications related to PBD and to surgery. The probabilities for patients in each group undergoing surgery, being resected if they did undergo surgery, and requiring a repeat laparotomy if they were resected were taken from a single large trial included in the Cochrane Review [17].

### 2.3 Outcomes

The quality of life component of QALYs is measured by utility scores. A utility score of 1 represents full health and a utility of 0 death; negative values represent states worse than death. A review of utility weights in the cost-effectiveness analysis registry [11] was undertaken using the search terms “pancreas,” “pancreatic,” “ampullary,” and “periampullary.” After reviewing the reference lists of the identified studies and removing duplicates, five studies containing potentially relevant utility data were identified [19–23]. The utility scores used in the model were from one study [22], selected because values were presented for different points over time and utility scores for all the health states in the model were included, thus enabling better comparability between values, and the values reported also reflected trends in disease-specific quality of life measures found in other studies (Table 2) [24–26]. Utility scores were measured at 6 wk, 3 mo, and 6 mo. QALYs were estimated using the trapezium rule for calculating the area under a curve. Because they did not measure directly the utility among patients undergoing PBD *versus* direct surgery for obstructive jaundice in patients with pancreatic and periampullary cancer, the utility scores were judged to be weak and so were tested comprehensively in sensitivity analyses.

### 2.4 Costs

The costs of PBD with major, minor, and no complications were assumed to be £4036, £2846, and £2897($5849, $4125, and $4199), respectively (Table 2), based on national mean costs of major endoscopic or percutaneous, hepatobiliary or pancreatic procedures provided on an elective inpatient basis [27]. Surgical resection with and without complications was assumed to cost £9209 ($13,346) and £7711 ($11,175), respectively [27]. Patients who undergo surgery but are not resected receive palliative surgery and this was assumed to cost £5378 ($7794) with complications and £4487 ($6503) without complications [27]. Patients who do not undergo surgery receive palliative treatment only, at an assumed cost of £4487 ($6503) [26]. The cost of repeat laparotomy in those who underwent surgical resection was assumed to be £7711 ($11,175) [27].

### 2.5 Measuring cost-effectiveness

Cost-effectiveness was measured using monetary net benefits (MNBs). For each treatment, the MNB was calculated as the mean QALYs per patient accruing to that treatment multiplied by decision makers' maximum willingness to pay for a QALY (also referred to as the cost-effectiveness threshold, which in the United Kingdom is approximately £20,000 [$28,986] to £30,000 [$43,478] per QALY gained) [28], minus the mean cost per patient for the treatment. This approach converts the outcomes from each treatment into monetary terms and then subtracts the costs of each treatment from the monetized benefits, calculating the net benefit of each treatment in monetary terms. MNBs were calculated using the base case parameter values shown in Table 2; these are referred to as deterministic results because they do not depend on chance. The treatment with the highest MNB represents the best value for money and is preferred on cost-effectiveness grounds.

### 2.6 Sensitivity analyses

One-way sensitivity analysis was undertaken, varying the probabilities, outcomes, and costs one at a time within the ranges listed in Table 2. The aim was to identify the threshold value for each parameter, where one exists, where the treatment with the highest MNB changed (e.g., the value at which PBD was the most cost-effective option). An analysis was also undertaken that based on the probabilities of complications with PBD and with complications of surgery on a single large trial [17] rather than all six studies included in the Cochrane Review.

A probabilistic sensitivity analysis (PSA) was undertaken, as recommended by the National Institute for Health and Care Excellence (NICE) [28]. Distributions were assigned to parameters (Table 2) to reflect the uncertainty with each parameter value. A random value from the corresponding distribution for each parameter was selected. This generated an estimate of the mean cost and mean QALYs and the MNB associated with each treatment. This was repeated 5000 times, and the results for each simulation were noted. The mean costs, QALYs, and MNBs for each treatment were calculated from the 5000 simulations; these are referred to as probabilistic results because they depend on chance. Using the MNBs for each of the 5000 simulations, the proportion of times each treatment had the highest MNB was calculated for a range of values for the maximum willingness to pay for a QALY. These were summarized graphically using cost-effectiveness acceptability curves [12].

In the PSA, we used beta and Dirichlet distributions to model uncertainty in the probabilities, beta distributions to model uncertainty in utility scores, and gamma distributions to model uncertainty in costs (Table 2) [29]. Dirichlet distributions were fitted using Excel macros developed by the Centre for Bayesian Statistics in Health Economics at the University of Sheffield [30]. In cases where standard errors were required for the PSA and these were not reported in the sources used, it was assumed the standard error was equal to the mean [29]. For the utilities, the variance was calculated assuming a beta distribution based on 97 observations [22,23]. Parameter values used to characterize each distribution are in Table 2. For each of the base case values, 95% confidence intervals (CIs) were derived using standard deviations calculated from the 5000 simulations in the PSA.

## 3 Results

Using base case values, PBD for obstructive jaundice in patients with pancreatic and periampullary cancer was more costly than direct surgery, with a mean cost per patient £10,775 (95%, CI £10,502 to £11,048, $15 616, 95% CI, $15 220 to $16 012) *versus* £8221 (95% CI, £7954 to £8487, $11 914, 95% CI, $11528 to $12 300); a significant cost increase of £2554 ($3701) per patient compared with direct surgery (Table 3). The increase in costs was due to the additional cost of the PBD procedure. QALYs up to 6 mo were slightly lower for PBD compared with direct surgery (0.337 [95% CI, 0.337–0.338] *versus* 0.343 [95% CI, 0.343–0.344]), because of the complications associated with PBD. The MNBs for PBD were significantly lower than those for direct surgery at maximum willingness to pay for a QALY of £20,000 ($28,986) and £30,000 ($43,478), indicating that direct surgery was preferred on cost-effectiveness grounds. As expected, the probabilistic results were numerically similar to the deterministic results (not shown).

In the one-way sensitivity analysis (Table 2), the results were neither sensitive to changing the values of the parameters within the ranges stated nor were they sensitive to basing the probabilities of complications with PBD and with complications of surgery on a single large trial [17]: in every situation direct surgery was the most cost-effective option.

**Fig. 2:**
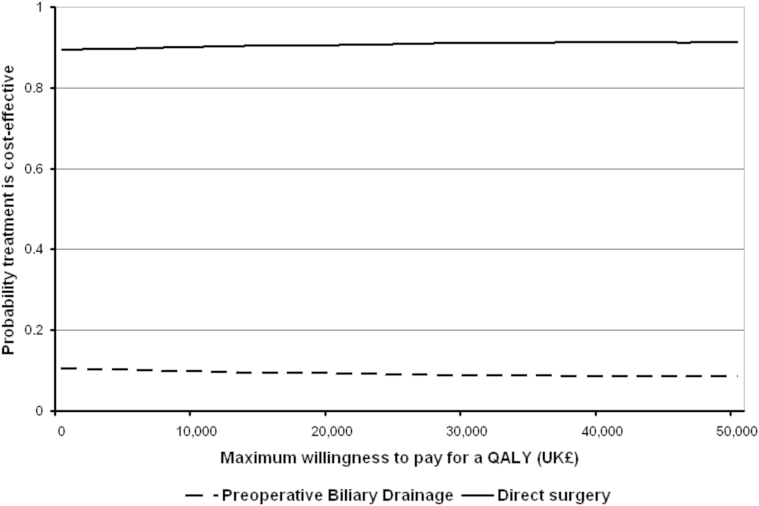
Cost-effectiveness acceptability curves showing the probability that each option is cost-effective at different values of the maximum willingness to pay for a QALY. In the United Kingdom, the lower and upper limit of the maximum willingness to pay for a QALY are £20,000 ($28 986) and £30,000 ($43 478), respectively.

The cost-effectiveness acceptability curves for each treatment show that PBD had a 9.5% probability of being cost-effective at a maximum willingness to pay for a QALY of £20,000 ($28,986) and a 8.9% probability at a value of £30,000 ($43,478; Fig. 2).

## 4 Discussion

### 4.1 Main findings

This study estimated the expected cost and QALYs of PBD *versus* direct surgery for obstructive jaundice in patients with pancreatic and periampullary cancer. Routine use of PBD was not cost-effective. It was more costly than direct surgery, with a mean cost per patient £10,775 ($15,616) *versus* £8221 ($11,914), respectively. It also produced fewer QALYs, with mean QALYs per patient of 0.337 *versus* 0.343, respectively. The MNB for PBD was lower than for direct surgery at a maximum willingness to pay for a QALY of £20,000 ($28,986) and £30,000 ($43,478). There is little uncertainty with this finding, demonstrated through extensive one-way and probabilistic sensitivity analyses.

### 4.2 Strengths and weaknesses

The strengths of this study are that it was based on a recently published Cochrane Review that analyzed in detail the available evidence for whether or not routine PBD is beneficial to patients with obstructive jaundice [8,9]. A comprehensive sensitivity analysis has also been performed, showing that although there is some uncertainty in the values used in the base case analysis, the conclusions are not sensitive to changing these values.

There are a number of weaknesses. First, the utility scores on which the QALY estimates were made are weak. However, the results are not sensitive to the values used, as demonstrated in sensitivity analyses. Second, the time horizon of the model over which costs and QALYs are measured is 6 mo. We have ignored differences in costs and QALYs beyond 6 mo. It may be that some of the complications of PBD persist beyond 6 mo, and if so including costs and outcomes beyond 6 mo would favor direct surgery. Third, the analysis was undertaken from the perspective of the UK NHS. A wider perspective, for example, a societal one, would also include impacts on the rest of society, including patients, families, and businesses. Given that PBD is associated with additional morbidity and also involves managing a drain during the period of PBD, it may be that if the costs from these other viewpoints were included, the cost increases attributable to PBD would be greater than shown.

### 4.3 Comparison with other studies

This is the first study to evaluate the cost-effectiveness of PBD for obstructive jaundice in pancreatic and periampullary cancer. The data on which this cost-effectiveness analysis was based were from a systematic review, which appraised the existing literature in depth [8,9].

### 4.4 Implications for policy and practice

This study provides a strong economic case to support the clinical evidence that PBD for obstructive jaundice in patients with pancreatic and periampullary cancer should not be used routinely. The findings are equally applicable in patients with distal cholangiocarcinomas and duodenal tumors with obstructive jaundice. This is because although the majority of tumors included in the trials, which provided the data for this cost-effectiveness analysis had pancreatic or ampullary tumors, the underlying reason for performing a PBD is the same in distal cholangiocarcinomas and duodenal tumors. These findings are applicable only in patients eligible for surgical resection with obstructive jaundice. Only about 20% of patients with pancreatic and periampullary cancer are eligible for surgical resection [1]. The findings are also not applicable in patients with cholangitis because of the common bile duct obstruction or in patients undergoing preoperative neoadjuvant chemotherapy. The mean duration of jaundice was stated in three trials and ranged between 28 and 55 d. So, the findings of this review are only applicable when the interval between jaundice and surgery is <2 mo on average [4,15,18]. However, our cost-effectiveness analysis provides a sound basis for avoiding excessive delays to surgery because of administrative reasons.

There is no evidence on the extent of use of PBD for obstructive jaundice in patients with pancreatic and periampullary cancer in the United Kingdom, so a national budget impact calculation is not possible. However, based on the findings in the present study, not performing PBD in patients with pancreatic and periampullary cancer would result in cost savings of approximately £2500 ($3623) per patient to the NHS.

### 4.5 Further research

This study is based on a Cochrane Review of PBD for obstructive jaundice. However, this analysis considered only the cost-effectiveness of PBD use in patients with resectable pancreatic and periampullary cancer who had been considered suitable for inclusion in trials comparing drainage with no drainage before surgery. Further economic evaluation is required to assess the costs and benefits in patients who were excluded from the trials such as those with cholangitis, high bilirubin levels (>250 μL/L), associated renal failure, or those undergoing preoperative neoadjuvant chemotherapy.

The Cochrane Review concluded that further randomized controlled trials, with low risk of bias, including long-term survival and quality of life measures are needed in patients with malignant obstructive jaundice. Such trials should also collect utility and cost data to facilitate cost-effectiveness analyses.

## 5 Conclusions

Routine PBD for obstructive jaundice in patients with pancreatic and periampullary cancer is not cost-effective.

## Figures and Tables

**Table 1:** Additional data extracted from randomized controlled trials included in the Cochrane Review [8].

Study	PBD				Direct surgery	
	Total number of patients	Number with minor complications related to PBD	Number with serious complications related to PBD	Complications of surgery (after PBD)	Total number of patients	Complications of surgery
Hatfield *et al.*[13]	29	NA	4	4	28	4
Lai *et al.*[14]	43	NA	12	16	44	18
McPherson *et al.*[15]	34	NA	8	9	31	13
Pitt *et al.*[16]	37	NA	4	16	38	20
van der Gaag *et al.*[17]	102	20	27	48	94	35
Wig *et al.*[18]	20	2	4	5	20	11
Total	265	22	59	98	255	101

NA = data not available.

**Table 2:** Model parameters for decision tree model and range of values used in univariate sensitivity analysis.

	Base case value	Distribution	Alpha	Beta	Sources	Range
Probabilities						
PBD						
Pr(major complications with PBD)	0.223	Dirichlet (59)			[13–18]	0–1
Pr(minor complications with PBD)	0.083	Dirichlet (22)			[13–18]	0–1
Pr(no complications with PBD)	0.694	Dirichlet (184)			[13–18]	0–1
Pr(undergoes surgery)	0.931	Beta	95	7	[17]	0–1
Pr(resected)	0.600	Beta	57	38	[17]	0–1
Pr(complications if resected)	0.370	Beta	98	167	[13–18]	0–1
Pr(complications if not resected)	0.370	Beta	98	167	[13–18]	0–1
Pr(repeat laparotomy)	0.211	Beta	12	45	[17]	0–1
Direct surgery						0–1
Pr(undergoes surgery)	0.979	Beta	92	2	[17]	0–1
Pr(resected)	0.685	Beta	63	29	[17]	0–1
Pr(complications if resected)	0.396	Beta	101	154	[13–18]	0–1
Pr(complications if not resected)	0.396	Beta	101	154	[13–18]	0–1
Pr(repeat laparotomy)	0.206	Beta	13	50	[17]	0–1
Unit costs						
PBD with major complications	4036 (5849)	Gamma	1	4036	[27]	2000–6500
PBD with minor complications	2846 (4125)	Gamma	1	2846	[27]	1700–3300
PBD without complications	2897 (4199)	Gamma	1	2897	[27]	1000–4000
Resection with complications	9209 (13,346)	Gamma	1	9209	[27]	6000–11,000
Resection without complications	7711 (11,175)	Gamma	1	7711	[27]	5000–10,000
Palliative surgery with complications	5378 (7794)	Gamma	1	5378	[27]	3500–6500
Palliative surgery without complications	4487 (6503)	Gamma	1	4487	[27]	2000–6000
Do not undergo surgery (palliative treatment only)	4487 (6503)	Gamma	1	4487	[27]	2000–6000
Repeat laparotomy	7711 (11,175)	Gamma	1	7711	[27]	5000–10,000
Utilities						
PBD with complications, undergoes surgery, resected, no complications						
6 wk	0.54	Beta	52.38	44.62	[22]	0–1
3 mo	0.74	Beta	71.78	25.22	[22]	0–1
6 mo	0.80	Beta	77.60	19.40	[22]	0–1
PBD with complications, undergoes surgery, resected, with complications						
6 wk	0.54	Beta	52.38	44.62	[22]	0–1
3 mo	0.71	Beta	68.87	28.13	[22]	0–1
6 mo	0.78	Beta	75.66	21.34	[22]	0–1
PBD with complications, undergoes surgery, not resected						
6 wk	0.54	Beta	52.38	44.62	[22]	0–1
3 mo	0.67	Beta	64.99	32.01	[22]	0–1
6 mo	0.72	Beta	69.84	27.16	[22]	0–1
PBD with complications, does not undergo surgery						
6 wk	0.54	Beta	52.38	44.62	[22]	0–1
3 mo	0.72	Beta	69.84	27.16	[22]	0–1
6 mo	0.72	Beta	69.84	27.16	[22]	0–1
PBD no complications, undergoes surgery, resected, no complications						
2 wk	0.60	Beta	58.20	38.80	[22]	0–1
3 mo	0.74	Beta	71.78	25.22	[22]	0–1
6 mo	0.80	Beta	77.60	19.40	[22]	0–1
PBD no complications, undergoes surgery, resected, with complications						
2 wk	0.60	Beta	58.20	38.80	[22]	0–1
3 mo	0.71	Beta	68.87	28.13	[22]	0–1
6 mo	0.78	Beta	75.66	21.34	[22]	0–1
PBD no complications, undergoes surgery, not resected						
2 wk	0.60	Beta	58.20	38.80	[22]	0–1
3 mo	0.67	Beta	64.99	32.01	[22]	0–1
6 mo	0.72	Beta	69.84	27.16	[22]	0–1
PBD no complications, does not undergo surgery						
2 wk	0.60	Beta	58.20	38.80	[22]	0–1
3 mo	0.72	Beta	69.84	27.16	[22]	0–1
6 mo	0.72	Beta	69.84	27.16	[22]	0–1
Direct surgery, undergoes surgery, resected, no complications						
2 wk	0.60	Beta	58.20	38.80	[22]	0–1
3 mo	0.74	Beta	71.78	25.22	[22]	0–1
6 mo	0.80	Beta	77.60	19.40	[22]	0–1
Direct surgery, undergoes surgery, resected, with complications						
2 wk	0.57	Beta	55.29	41.71	[22]	0–1
3 mo	0.71	Beta	68.87	28.13	[22]	0–1
6 mo	0.78	Beta	75.66	21.34	[22]	0–1
Direct surgery, undergoes surgery, not resected						
2 wk	0.54	Beta	52.38	44.62	[22]	0–1
3 mo	0.67	Beta	64.99	32.01	[22]	0–1
6 mo	0.72	Beta	69.84	27.16	[22]	0–1
Direct surgery, does not undergo surgery						
2 wk	0.76	Beta	73.72	23.28	[22]	0–1
3 mo	0.72	Beta	69.84	27.16	[22]	0–1
6 mo	0.72	Beta	69.84	27.16	[22]	0–1

Unit costs are in 2011–2012 UK£ (US$). The base case values are used to produce the deterministic results. The distributions are used to undertake the probabilistic sensitivity analysis, to produce the probabilistic results, and construct the cost-effectiveness acceptability curves.

**Table 3:** Base case results.

	PBD		Direct surgery	
Costs				
UK£	10,775	(10,502 to 11,048)	8221	(7954 to 8487)
US$	15,616	(15,220 to 16,012)	11,914	(11,528 to 12, 300)
QALYs	0.337	(0.337 to 0.338)	0.343	(0.343 to 0.344)
MNB				
UK£20,000	−4031	(−3758 to −4304)	−1359	(−1092 to −1626)
US$28 986	−5843	(−6485 to −5685)	−1969	(−2551 to –1768)
UK£30,000	−659	(−386 to −933)	2072	(1805 to 2340)
US$43 478	−956	(−1599 to –798)	3003	(2424 to 3206)

Costs are in 2011–2012 UK£ and US$. Figures are expected values per patient with 95% CIs in brackets. The point estimates are calculated using base case values of the model parameters (deterministic results). The 95% CIs are derived using standard deviations calculated from the 5000 simulations in the probabilistic sensitivity analysis. The MNB is calculated at a maximum willingness to pay for a QALY of £20,000 ($28 986) and £30,000 ($43 478). Numbers may not sum because of rounding.
